# Chromatin dynamics and in vitro biomarkers in laminopathies: an overview

**DOI:** 10.1186/1750-1172-10-S2-O12

**Published:** 2015-11-11

**Authors:** Giovanna Lattanzi

**Affiliations:** 1 CNR Institute for Molecular Genetics, Unit of Bologna and Rizzoli Orthopedic Institute, Laboratory of Musculoskeletal Cell Biology, Bologna, Italy

## 

Chromatin regulation in eukaryotes occurs through complex and interconnected mechanisms that ensure heterochromatin maintenance and compartmentalization of chromosome domains, genome stability, chromatin conformational changes before and after mitosis, gene silencing and transcriptional activation and chromatin remodeling at specific promoters. We refer to these events as a whole using the term “chromatin dynamics.” Chromatin dynamics involves a number of protein families including epigenetic enzymes, DNA repair factors, heterochromatin proteins, transcription factors and transcriptional regulators. Although lamins have been involved in almost all the processes that regulate chromatin dynamics[1], three main functions link lamins to chromatin regulation: recruitment of the DNA damage response proteins[2], transcription factor binding[3,4] and modulation and maintenance of heterochromatin domains[5]. Our preliminary data have shown that lamin A/C plays a major role in anchorage of epigenetic enzymes in nuclei and loss of lamin A/C- histone deacetylase (HDAC) binding, as occurs in Hutchinson-Gilford progeria (HGPS) cells, affects enzyme activity and histone acetylation. These results may explain our previously published data[6] showing that the heterochromatin defects of HGPS cells can be rescued by combined inhibition of prelamin A farnesylation and HDAC activity and pave the way to new therapeutic perspectives. Moreover, altered lamin A/C-HDAC interaction and histone acetylation patterns can be explored as potential biomarkers for laminopathies.
